# *Circ_0006476* modulates macrophage apoptosis through the miR-3074-5p/DLL4 axis: implications for Notch signalling pathway regulation in cardiovascular disease

**DOI:** 10.18632/aging.206049

**Published:** 2024-08-19

**Authors:** Lin Cong, Lili Zhao, Ying Shi, Yunpeng Bai, Zhigang Guo

**Affiliations:** 1 Academy of Medical Engineering and Translational Medicine, Tianjin University, Tianjin, China; 2 Tianjin Institute of Cardiovascular Diseases, Chest Hospital, Tianjin University, Tianjin, China; 3 Department of Cardiac Surgery, Chest Hospital, Tianjin University, Tianjin, China; 4 Clinical School of Thoracic, Tianjin Medical University, Tianjin, China; 5 Tianjin Key Laboratory of Cardiovascular Emergency and Critical Care, Tianjin Municipal Science and Technology Bureau, Tianjin, China

**Keywords:** apoptosis, *circ_0006476*, *miR-3074-5p*, *DLL4*, Notch signalling pathway

## Abstract

As the population ages, the prevalence of atherosclerosis (AS), a significant cause of cardiovascular disease (CVD), continues to increase. Apoptosis is an independent risk factor for atherosclerosis. Macrophages are the primary immune cell group in AS lesions, and their apoptosis plays a crucial role in the occurrence and development of AS. There is a common mechanism of action for circular RNAs (circRNAs) that involves the sponging of microRNAs (miRNAs) by binding to the miRNA response element (MRE), thereby increasing the transcription of their target messenger RNAs (mRNAs). Most diseases are profoundly reliant on circRNAs. However, the underlying mechanism of circRNAs in apoptosis is yet to be elucidated. All differentially expressed genes (DEGs) and their expression levels were analysed by whole-transcriptome sequencing of samples from the control and nicotine groups of THP-1 macrophages. GO and KEGG analyses revealed that nicotine affects macrophage physiological processes and related pathways. GSEA focused on gene sets to better understand the potential pathways and biological functions of all mRNAs. A competitive endogenous RNA (ceRNA) regulatory network was constructed and validated through molecular biology experiments. The Notch signalling pathway was activated in nicotine-treated macrophages, and the expression of DLL4 in this pathway was increased. *Circ_0006476 *is involved in apoptosis via *miR-3074-5p*/*DLL4*, regulating pathogenic processes related to the Notch signalling pathway. The better we understand the pathways involved in macrophage apoptosis, the more likely we are to find other novel therapeutic targets that can help treat, prevent, and reduce the mortality associated with AS.

## INTRODUCTION

Atherosclerosis (AS) is a chronic inflammatory condition of the arterial wall characterized by the accumulation of lipids, inflammation, and apoptosis, and it is the primary cause of cardiovascular disease (CVD) [1, 2]. According to the China Cardiovascular Health and Disease Report 2021, CVD is the leading cause of both illness and death in China, affecting an estimated 330 million individuals [3]. As a crucial element within inflammatory plaques, macrophages play a pivotal role in the development of atherosclerotic cardiovascular disease (ASCVD) [4, 5]. A growing body of research indicates that macrophage apoptosis is a critical factor in determining the stage of AS lesions and the stability of plaques. In the early stages of atherosclerotic lesions, lipid-laden macrophages undergo apoptosis and are effectively removed through efferocytosis [6]. However, in advanced atherosclerotic lesions, apoptotic cells are not efficiently cleared, leading to inflammation and the formation of necrotic cores [6].

The pivotal role of Notch signalling in apoptosis is widely recognized and evolutionarily conserved [7]. Activation of Notch signalling occurs through intramembrane proteolysis mediated by γ-secretase, leading to cleavage of the Notch intracellular domain (NICD) when ligands (including Jagged1, Jagged2, Delta-like DLL1, DLL3, and DLL4) bind to their specific receptors (Notch 1-4) [8]. Subsequently, the NICD translocates to the nucleus, where it typically forms a complex with transcription factors and coactivators, ultimately facilitating the transcription of target genes—a well-documented process. Among the well-known targets of Notch signalling are Hey1, Hey2 and L from the Hey family and Hes1-7 from the Hairy/Enhancer of Split family [8]. It is widely recognized that components of the Notch signalling pathway are frequently subject to mutations and exhibit dysregulated expression in various types of AS and other CVDs [9].

Until now, it has been hypothesized that smoking might be associated with an unfavourable prognosis for diseases, given the substantial amount of data highlighting the detrimental impact of smoking on cardiovascular health and its connection to various CVDs [10]. Nicotine, a primary constituent of cigarette smoke and one of the most pharmacologically active components [11], has recently emerged as a pivotal regulator of immune cell apoptosis or programmed cell death (PCD) [12]. Nevertheless, the precise mechanism by which nicotine exacerbates CVD remains unclear. Previous research has revealed that nicotine induces apoptosis in cardiomyocytes by triggering oxidative stress and altering gene expression patterns related to apoptosis [13].

A novel noncoding RNA known as a circRNA is predominantly found in the cytoplasm, indicating its role in posttranscriptional regulation [14]. This type of circRNA contains numerous microRNAs (miRNAs) and their binding sites, referred to as miRNA response elements (MREs), which enable them to function as miRNA sponges or competing endogenous RNAs (ceRNAs) [15]. CeRNAs have emerged as significant biomarkers, therapeutic targets, and prognostic indicators. It has promising clinical applications in CVD treatment, such as myocardial hypertrophy, myocardial infarction, and AS, indicating that it has excellent research value [16]. Previous studies have demonstrated that circRNAs primarily regulate the progression of CVDs by functioning as ceRNAs, thereby modulating downstream messenger RNA (mRNA) expression through their miRNA sponge effect [17–19]. However, the specific expression profile of circRNAs and their role in macrophage apoptosis have remained unclear. In this study, we performed whole-transcriptome sequencing analysis of THP-1 macrophage samples treated with different methods to elucidate the potential mechanism of the circRNA-mediated ceRNA regulatory network in the development of macrophage apoptosis. This analysis revealed the quantity and expression levels of DEcircRNAs, DEmiRNAs, and DEmRNAs in nicotine-treated macrophages compared to those in normal macrophages. Based on sequencing data, we predicted the circRNA–miRNA–mRNA network regulated by nicotine through correlation coefficient and base complementary pairing analysis. We conducted GO and KEGG pathway enrichment analyses on all the DEcircRNA parental genes and DEmRNAs, enabling us to better understand their roles in biological processes and molecular pathways. The GSEA method was used to identify critical metabolic and signalling pathways in the disease development process by identifying gene sets in the expression matrix of all genes and considering genes with subtle variations. Based on sequencing data, we constructed a putative circRNA miRNA–mRNA regulatory network involved in macrophage apoptosis and conducted molecular biology validation.

## MATERIALS AND METHODS

### Cell culture

Human THP-1 monocytes (Procell Life Technology Co., Ltd., Wuhan, China) were cultured in complete medium consisting of RPMI 1640 medium (Gibco, USA) supplemented with 10% fetal bovine serum (FBS; Gibco, USA). The cells were maintained at 37°C with 5% CO_2_, and the medium was replaced every 2–3 days. To induce differentiation into macrophages, aliquots of THP-1 monocytes (1.0 × 10^5^ cells/ml) were added dropwise to six-well plates in complete medium supplemented with 100 ng/ml phorbol-12-myristate-13-acetate (PMA; Sigma, USA). During this process, cells undergo morphological and growth mode changes, transitioning from suspended growth to adherent growth and from a circular to irregular shape, with further increase in volume. The cytoplasm became loose, the number of nuclei increased significantly, and many distinct organelles were visible. A small amount of protrusions can be seen around the cell membrane. The above phenomenon indicates that THP-1 monocytes are induced to differentiate into undifferentiated M0 macrophages.

### RNA extraction, library construction, and sequencing

Total RNA was extracted from a TRIzol kit (Invitrogen, USA), and then quality inspection was carried out by three methods (agarose gel electrophoresis, NanoDrop micro spectrophotometer detection, and Agilent 2100 detection). Fragment buffer was used to convert enriched mRNA fragments into short fragments, and the fragments were passed through a Hieff NGS^®^ Ultima Dual mode mRNA Library Prep Kit for reverse transcription into cDNA. Gene Denovo Biotechnology Co.’s Illumina HiSeq^™^ 4000 was used to sequence the obtained cDNA library.

### DEcircRNAs, DEmiRNAs and DEmRNAs

The software DESeq2 was used to assess differences in gene expression between two separate groups. mRNAs with a false discovery rate (FDR) <0.05 and a |fold change (FC)|>1.5 were considered differentially expressed mRNAs (DEmRNAs). CircRNAs with an FDR <0.05 and a |FC|>2 were deemed to be differentially expressed (DEcircRNAs). Differentially expressed miRNAs (DEmiRNAs) were defined as those with an FDR <0.05 and a |FC|>1.5. In the R environment, ggplot2 was used to construct volcano maps of differentially expressed genes (DEGs) in two sets of samples.

### Gene ontology (GO)

First, all DEGs were mapped to the Gene Ontology (GO) term in the Gene Ontology database (http://www.geneontology.org/), the number of genes associated with each term was calculated, and the GO terms associated with DEGs that were significantly enriched compared to the genomic background were defined through hypergeometric testing. The formula for calculating the *P*-value is as follows:


$$p=1-\sum_{i=0}^{m-1}\frac{\left(\begin{array}{c} M\\ i \end{array}\right)\left(\begin{array}{c} N-M\\ n-i \end{array}\right)}{\left(\begin{array}{c} N\\ n \end{array}\right)}$$

Here, *N* is the number of genes with GO annotation, *n* is the number of DEGs in *N*, *M* is the number of all genes annotated to a specific GO term, and *m* is the number of DEGs in *M*. The calculated *p*-value is corrected by FDR, with FDR <0.05 as the threshold. GO terms that met these criteria were defined as those significantly enriched in DEGs.

### Pathway enrichment analysis

Kyoto Encyclopedia of Genes and Genomes (KEGG) enrichment analysis is the main public pathway-related database. Compared with the whole-genome background, KEGG enrichment analysis revealed significantly enriched metabolic or signalling pathways among the DEGs. The calculation formula was the same as that for the GO analysis.

### Gene set enrichment analysis (GSEA)

We conducted gene set enrichment analysis (GSEA) using the MSigDB database to determine differences between the two groups in terms of specific GO terms, KEGG pathways, Reactome pathways, and DO terms. The gene expression matrix was input, and the genes were ranked using the signal-to-noise normalization method. It is generally believed that a gene set under pathways with *p* < 0.05, |NES|>1, and FDR <0.25 is meaningful.

### miRNA target prediction

Based on the results of the differential expression analysis, we selected target miRNAs for target gene prediction and searched for potential target genes regulated by these miRNAs. We used the miRanda and TargetScan methods for target gene prediction, and the intersection was used as the prediction result.

### Construction of the ceRNA network

A ceRNA network based on the ceRNA theory was constructed utilizing the Spearman rank correlation coefficient (SCC). (1) Pairs with SCCs less than 0.7 were identified as negatively coexpressed circRNA-miRNA or mRNA–miRNA pairs, with both mRNAs and circRNAs serving as miRNA target genes and differentially expressing all RNAs. (2) By utilizing the Pearson correlation coefficient (PCC), we gauged the correlation between circRNA and mRNA expression. Pairs with a PCC greater than 0.9 were chosen as coexpressed circRNA-mRNA pairs, with both mRNAs and circRNAs in this pair being targeted and coexpressed inversely with a shared miRNA. (3) We employed a hypergeometric cumulative distribution function test for samples to ascertain whether the miRNA sponges shared between the two genes were meaningful. Only those gene pairs with a *p*-value less than 0.05 were chosen.

### Quantitative real-time PCR (qPCR)

Using the UNIQ-10 Columnar Total RNA Extraction Kit (Sangon, China), mRNA extraction was conducted, and reverse transcription was performed with the RT Easy II First Strand cDNA Synthesis Kit. Subsequently, (TIANGEN, China), 2 μL of cDNA was amplified using Real-Time PCR Easy on an ABI 7500 Fast Real-Time System (GAPDH was used as an internal reference).

Using the miRcute Plus miRNA Isolation Kit for miRNA extraction (TIANGEN, China), reverse transcription was conducted with the miRcute Plus miRNA First-Strand Kit (TIANGEN, China). Subsequently, 1 μL of cDNA was amplified with the miRcute Plus miRNA qPCR Kit on an ABI 7500 Fast Real-Time System (U6 was used as an internal reference).

CircRNA extraction was accomplished with the UNIQ-10 Columnar Total RNA Extraction Kit (Sangon, China), while reverse transcription was accomplished with the riboSCRIPT mRNA/lncRNA qPCR Starter Kit (Ribo Biotech, China). Then, 2 μL of cDNA was amplified using the riboSCRIPT mRNA/lncRNA qPCR Starter Kit on an ABI 7500 Fast Real-Time System (GAPDH was used as an internal reference). The primer sequences are displayed in Table 1.

**Table 1:** Primer sequences.

**Species**	**Gene**	**Forward primer**	**Reverse primer**
Human	*Bax*	CCCGAGAGGTCTTTTTCCG	GCCTTGAGCACCAGTTTGC
Human	*BCL2*	GGTGGACAACATCGCTCTG	ACAGCCAGGAGAAATCAAACA
Human	*DLL4*	GTCTCCACGCCGGTATTGG	CAGGTGAAATTGAAGGGCAGT
Human	*GAPDH*	AACGGATTTGGTCGTATTG	GCTCCTGGAAGATGGTGAT
Human	*circ_0006476*	AAACTCACACAGAGGAGAAGC	ATTTCTGCCATTTGTATGCCGT
Human	*miR-3074-5p*	GTTCCTGCTGAACTGAGCCAG
Human	*miR-30-X*	GCGTGTAAACATCCTTGACTGGAAGCG	
Human	*miR-3944-X*	TGTGCAGCAGGCCAACCGAGAAT
Human	*miR-9985-Z*	GCCGGCGTTCACAGTGGCTAATTT	
Human	*U6*	CTCGCTTCGGCAGCACA	AACGCTTCACGAATTTGCGT

### Western blot analysis (WB)

After being subjected to different treatments, the THP-1 macrophages were lysed in RIPA buffer (Beyotime, China). After electrophoresis and membrane transfer, the membranes were blocked with 5% skim milk powder (Solarbio, China) at room temperature for 1.5 hours. Primary antibodies (DLL4, HES1, NICD, Bcl-2, BAX, and GAPDH) were diluted overnight at 1:1000 in 5% BAS-TBST buffer (Solarbio, China) at 4°C. Subsequently, the cells were incubated with an HRP-conjugated secondary antibody (1:5000) for 1.5 hours. An imaging system (Bio-Rad, USA) was used for chemiluminescence-mediated immunoblotting. The WB results were quantified using Quantity One software.

### Flow cytometry

An Annexin V-FITC/PI cell apoptosis detection kit (Absin, China) was used to measure the percentage of nicotine-induced THP-1 macrophage apoptosis using Annexin V-FITC and propidium iodide (PI) staining. Pretreated cells were collected after digestion with trypsin, washed with PBS three times, and then stained with Annexin V-FITC and PI in the dark for 15 minutes. Flow cytometry was used to detect apoptotic cells at different stages.

### Transferase dUTP nick end labelling (TUNEL) assay

A One Step TUNEL Apoptosis Assay Kit (Beyotime, China) was used for TUNEL staining. The cells were exposed to 0 or 1 μM nicotine for the same period. Subsequently, the cells were fixed with 4% paraformaldehyde. Following the washes, the cells were incubated in a 0.1% Triton X-100 PBS permeabilization solution. Subsequently, the samples were incubated in 50 μl of TUNEL reaction mixture for 1 h in the dark. Fluorescence microscopy was used to image the samples.

### Hoechst assay

Following the instructions of Beyotime Biotechnology, the cells were fixed, washed twice, and then stained with Hoechst staining solution (Beyotime, China). The nuclei were observed under a Nikon fluorescence microscope.

### Transfection of small interfering RNA (siRNA)

The mixture consisted of 2 μL of siRNA (GenePharma, China) (Si-*DLL4, miR-3074-5p* inhibitor, and Si-*circ_0006476*) and 5 μL of Hieff TransTM siRNA (Yeason, China), which were added to 200 μL of RPMI 1640 medium (Gibco, USA). Following the combination, the mixture was carefully introduced into individual macrophage cultures in a plate dish along with medium. The cultures were then incubated undisturbed for a period of 48 h. Interference fragments are shown in Table 2.

**Table 2:** siRNA duplexes.

**Gene**	**Sense**	**Antisense**
*DLL4* S1	GUGGGUCAGAACUGGUUAUTT	AUAACCAGUUCUGACCCACTT
*DLL4* S2	GGCCAACUAUGCUUGUGAATT	UUCACAAGCAUAGUUGGCCTT
*DLL4* S3	CAGUCUGUGUGUUUGAUAUTT	AUAUCAAACACACAGACUGTT
*miR-3074-5p* inhibitor	CUGGCUCAGUUCAGCAGGAAC	
*circ_0006476* S1	AUGUAAGAAUGGCAAGUUAAATT	UUUAACUUGCCAUUCUUACAUTT
*circ_0006476* S2	GAAUGUAAGAAUGGCAAGUUATT	UAACUUGCCAUUCUUACAUUCTT
*circ_0006476* S3	CCCUUUGAAUGUAAGAAUGGCTT	GCCAUUCUUACAUUCAAAGGGTT

### RNA pulldown assay

An RNA pulldown assay was performed in THP-1 macrophages using an RNA pulldown kit (BersinBio, China) according to the manufacturer’s instructions. Briefly, a biotin-labelled *circ_0006476* probe (BersinBio, China) or negative control probe (BersinBio, China) was incubated with streptavidin magnetic beads. THP-1 macrophages were collected and lysed in RIP buffer to obtain total protein extracts. The probe magnetic beads and cell extract were mixed and washed with NT2 buffer. Proteins bound to the probe were eluted with protein elution buffer and subjected to WB.

### Statistical analysis

The data are presented as the means±standard deviations (SD), and significant differences between the two groups were determined using Student’s *t*-test. For comparisons involving more than two groups, ANOVA followed by the Tukey post hoc test was used to assess statistical significance. A *p*-value less than 0.05 was considered to indicate statistical significance. Statistical analyses were performed using GraphPad Prism 5 software.

### Availability of data and materials

The datasets generated and analysed during the current study are available in the Sequence Read Archive (SRA) repository, PRJNA1077755.

## RESULTS

### Nicotine induces pyroptosis in THP-1 macrophages

**Figure 1:**
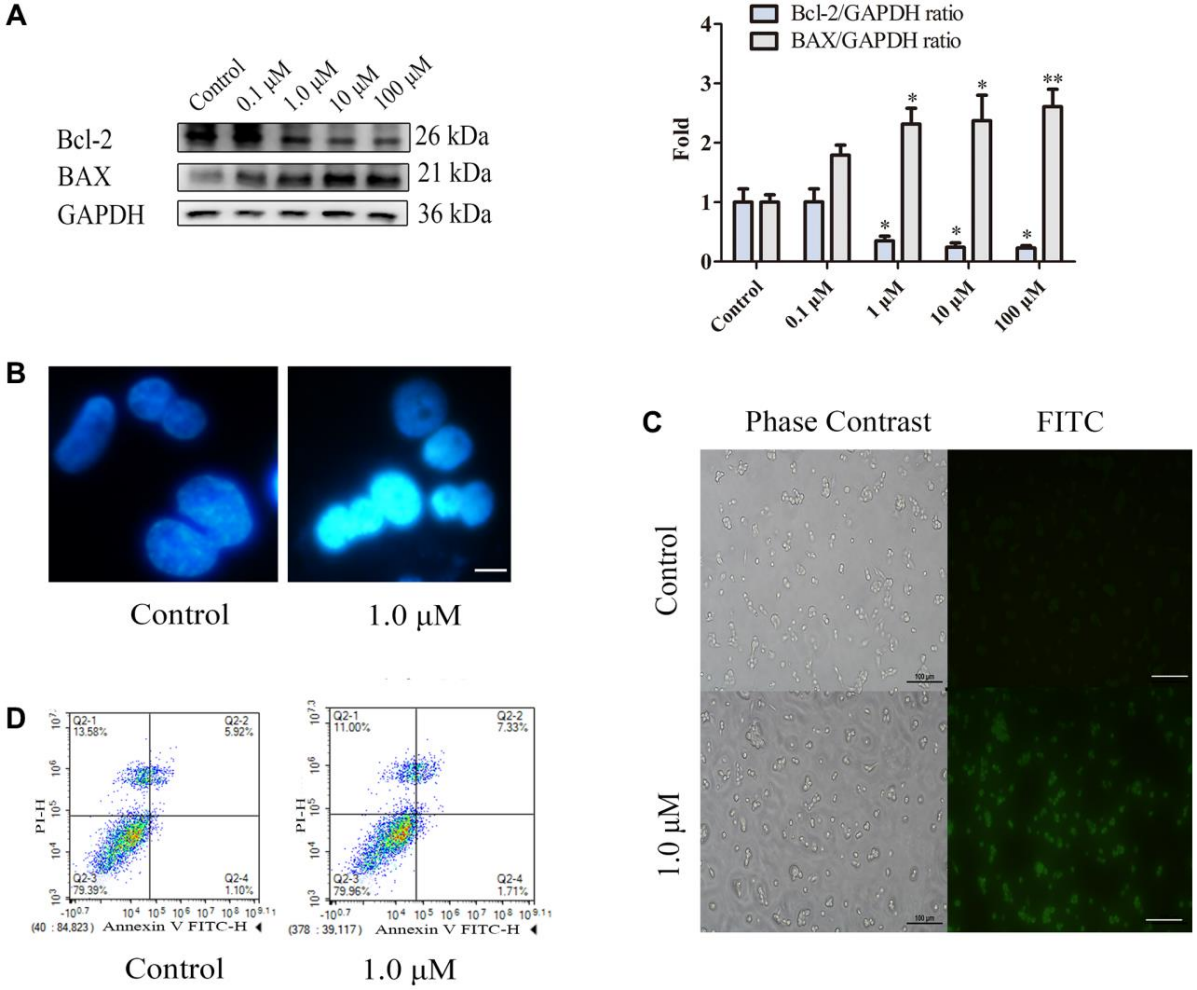
**Nicotine induces apoptosis in THP-1 macrophages.** (**A**) Dose-dependent expression of apoptosis-related proteins in THP-1 macrophages (24 h of exposure) analysed by WB. (**B**) Hoechst staining of THP-1 macrophages (bright blue represents the apoptotic cell nucleus) (scale bar: 50 μm). (**C**) TUNEL staining was used to evaluate the number of apoptotic THP-1 macrophages (green fluorescence indicates apoptotic cells) (scale bar: 50 μm). (**D**) Flow cytometry assay measuring apoptotic and necrotic cell levels. (*n* = 3. The data are presented as the mean ± SD. ^*^P < 0.05, ^**^P < 0.01, and ^***^P < 0.001 vs. the control group).

The BAX protein plays a vital role in regulating apoptosis, as it is one of the upstream proteins involved in the initiation of apoptosis. Moreover, the antiapoptotic effect of the Bcl-2 protein is achieved by antagonizing the BAX protein, inhibiting the release of Cyt-c from mitochondria to the cytoplasm and preventing the activation of the apoptosis-related protein Caspase-3. Therefore, we used WB to detect the effects of different nicotine concentrations on the expression levels of the apoptosis-related proteins BAX and Bcl-2 in THP-1 macrophages. As shown in Figure 1A, the WB results showed that the expression levels of apoptosis-related proteins changed in a concentration-dependent manner (24 h). The expression of the antiapoptotic protein Bcl-2 was significantly reduced (*P* < 0.05), and the expression of the proapoptotic protein BAX was significantly increased (*P* < 0.05) in the 1 μM nicotine group. Therefore, the WB results indicated that treatment of macrophages with 1 μM nicotine for 24 hours increased the number of apoptotic cells. Hoechst is a membrane-permeable fluorescent dye that can bind to DNA double helix grooves, especially in regions rich in AT. Therefore, both normal cells and early apoptotic cells can be stained with Hoechst. The normal nucleus was circular, with evenly distributed DNA and uniform blue staining. The apoptotic cell nucleus looks like a crescent or fragmented, and after concentrated staining, it appears as a bright blue solid or clumped shape, or the nucleus is divided into leaves, fragments, and edges. Hoechst staining of THP-1 macrophages treated with nicotine revealed condensed bright nuclei typical of apoptotic dead cells (bright blue represents the apoptotic cell nucleus), with almost no apoptotic nuclei observed in control macrophages (Figure 1B). TUNEL staining is a standard method for detecting apoptosis in cells. In principle, some DNA endonucleases are activated when cells undergo apoptosis, which cuts off genomic DNA between nucleosomes. During cell apoptosis and genomic DNA breakage, the exposed 3′-OH can be catalyzed by terminal deoxynucleotide transferase to add fluorescein and biotin-labelled dUTP, allowing for the observation of green fluorescence through fluorescence microscopy. Moreover, we assessed apoptosis in nicotine-treated THP-1 macrophages using terminal deoxynucleotidyl TUNEL staining, which revealed an increase in TUNEL-positive cells (green fluorescence represents apoptotic cells) in the THP-1 macrophages treated with 1 μM nicotine compared to the control (Figure 1C). Annexin V and PI dual staining is a classic flow cytometry method for detecting cell apoptosis. In the early stage of apoptosis, phosphatidylserine (PS) flips from the inner side of the cell membrane to the surface of the cell membrane. Annexin V is a Ca^2+^-dependent phospholipid-binding protein with a molecular weight of 35–36 kDa. It can bind with PS with high affinity. Annexin V is labelled with fluorescein isothiocyanate (FITC), and the occurrence of cell apoptosis can be detected using flow cytometry or fluorescence microscopy. PI is a dye that can bind to DNA and cannot penetrate the complete cell membrane of regular or early apoptotic cells. However, in the late stage of apoptosis and in dead cells, PI can penetrate the cell membrane and cause red staining of the nucleus. Therefore, we used conventional flow cytometry techniques labelled with FITC and PI to determine the percentage of early and late apoptotic cells in nicotine- and MT-treated cultures. Early apoptotic cells were identified by the Annexin V-FITC^+^/PI^−^ staining pattern, while late apoptotic cells exhibited the Annexin V-FITC^+^/PI^+^ staining pattern. Flow cytometry revealed a significant increase in the number of early and late apoptotic cells after 1 μM nicotine treatment compared to that in untreated control cell cultures (Figure 1D). These findings collectively support the conclusion that nicotine reduces cell viability through apoptotic mechanisms, and subsequent experiments were performed to induce apoptosis in THP-1 macrophages by applying 1 μM nicotine for 24 h.

### Differentially expressed genes related to nicotine-treated macrophages

**Figure 2:**
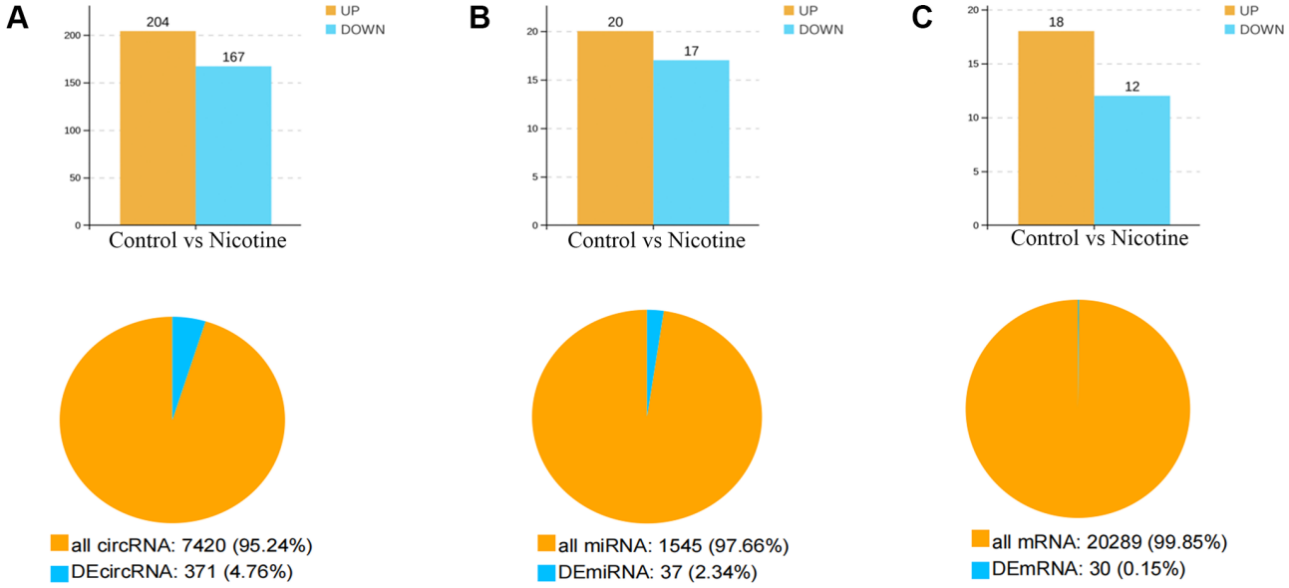
**Number and expression of differentially expressed genes.** (**A**) Quantification and expression of DEcircRNAs (|FC|>2, *P* < 0.05). (**B**) Quantification and expression of DEmiRNAs (|FC|>1.5, *P* < 0.05). (**C**) Quantity and expression of DEmRNAs (|FC|>1.5, *P* < 0.05).

Whole-transcriptome sequencing was conducted on six samples, which were divided into two groups: control cells and nicotine-treated cells. This analysis provided a comprehensive overview of RNA quantity and expression levels. Differential expression analysis revealed significant differences in the expression of circRNAs, miRNAs, and mRNAs between the nicotine and control groups. Specifically, Figure 2A–2C illustrates the findings, which included 371 DEcircRNAs (204 upregulated and 167 downregulated, accounting for 4.67% of the total circRNAs) with |FC|>2 and *P* < 0.05, 37 DEmiRNAs (20 upregulated and 17 downregulated, accounting for 2.34% of the total miRNAs) with |FC|>1.5 and *P* < 0.05, and 30 DEmRNAs (18 upregulated and 12 downregulated, accounting for 0.15% of the total mRNA count) with |FC|>1.5 and *P* < 0.05.

Table 3 presents the top 30 DEcircRNAs with the most significant upregulation and downregulation changes. All circRNAs within the top 30 were upregulated, with hsa_circ_0030069 exhibiting the most significant upregulation. Table 4 lists the 30 DEmiRNAs displaying the most pronounced upregulation and downregulation patterns. Among these, hsa-miR-6747-3p was the miRNA with the most significant upregulation, while hsa-miR-206 showed the most significant downregulation. Table 5 shows the 30 DEmRNAs with the greatest upregulation or downregulation. ENSG00000256407 was the mRNA that displayed the most significant upregulation, while ENSG00000283149 exhibited the most significant downregulation.

**Table 3:** DEcircRNAs.

**Id**	**Log2 (FC)**	***P*-value**	**circBase ID**	**Regulation**
ENSG00000172766	19.06135514	7.65E-06	hsa_circ_0030069	UP
ENSG00000274070	18.46972573	0.000488819	–	UP
ENSG00000112640	18.46972573	0.000488819	hsa_circ_0076437	UP
ENSG00000134802	18.46972573	0.000488819	hsa_circ_0022132	UP
ENSG00000175198	18.19335738	0.00195459	hsa_circ_0030754	UP
ENSG00000109323	18.16027292	0.00195459	hsa_circ_0001432	UP
ENSG00000145216	18.14779764	0.003908594	hsa_circ_0069737	UP
ENSG00000105246	18.14779764	0.003908594	–	UP
ENSG00000077420	18.06135514	0.003908594	hsa_circ_0017997	UP
ENSG00000198431	18.04384835	0.003908594	hsa_circ_0027960	UP
ENSG00000171735	17.96503518	0.003908594	hsa_circ_0009542	UP
ENSG00000133961	17.95515256	0.007816146	hsa_circ_0008406	UP
ENSG00000198131	17.95515256	0.007816146	hsa_circ_0000967	UP
ENSG00000149311	17.94075341	0.007816146	hsa_circ_0024229	UP
ENSG00000152359	17.92620909	0.007816146	hsa_circ_0073053	UP
ENSG00000141298	17.92620909	0.007816146	hsa_circ_0042820	UP
ENSG00000175198	17.73276014	0.015630469	hsa_circ_0030754	UP
ENSG00000139324	17.73276014	0.015630469	hsa_circ_0027691	UP
ENSG00000162772	17.73276014	0.015630469	–	UP
ENSG00000137767	17.73276014	0.015630469	–	UP
ENSG00000096717	17.73276014	0.015630469	hsa_circ_0018471	UP
ENSG00000173064	17.73276014	0.015630469	–	UP
ENSG00000089248	17.73276014	0.015630469	–	UP
ENSG00000133624	17.73276014	0.015630469	–	UP
ENSG00000122218	17.73276014	0.015630469	hsa_circ_0014908	UP
ENSG00000135919	17.73276014	0.015630469	hsa_circ_0008365	UP
ENSG00000114062	17.71594709	0.015630469	hsa_circ_0034165	UP
ENSG00000105939	17.71594709	0.015630469	hsa_circ_0082622	UP
ENSG00000152061	17.68172151	0.015630469	hsa_circ_0015334	UP
ENSG00000102921	17.66429935	0.015630469	hsa_circ_0039316	UP

**Table 4:** DEmiRNAs.

**Id**	**Log2 (FC)**	***P*-value**	**Regulation**
hsa-miR-206	−6.862038047	0.002496363	DOWN
hsa-miR-6747-3p	5.742275748	0.008604406	UP
hsa-miR-586	5.70016225	0.006725859	UP
miR-107-z	−5.374459919	0.04231448	DOWN
miR-885-y	5.318557873	0.026137781	UP
miR-3944-x	−5.12983251	0.021967984	DOWN
hsa-let-7a-2-3p	−3.673119112	0.008073136	DOWN
miR-4466-z	−3.275503758	0.036881144	DOWN
miR-326-z	3.156659061	0.007687275	UP
miR-9985-z	−2.874431932	0.01120557	DOWN
miR-196-y	2.645873775	0.03802994	UP
hsa-miR-3126-5p	−2.599843448	0.049289742	DOWN
novel-m0142-5p	−2.499521252	0.022049291	DOWN
miR-218-x	−2.48577105	0.023702183	DOWN
novel-m0118-3p	2.369243411	0.025500452	UP
hsa-miR-1260a	−2.338655004	0.026991179	DOWN
hsa-miR-3128	−2.001403855	0.026759362	DOWN
hsa-miR-10b-5p	1.852745152	0.014738408	UP
miR-21-z	−1.812351653	0.017354613	DOWN
hsa-miR-150-5p	1.749865396	0.010573319	UP
hsa-miR-3074-5p	−1.656512816	0.013318925	DOWN
miR-188-x	1.598706578	0.041051289	UP
hsa-miR-486-5p	1.48383397	0.035917054	UP
novel-m0037-3p	1.458954285	0.043017226	UP
hsa-miR-451a	1.456351917	0.008826723	UP
novel-m0039-3p	1.448933074	0.035806501	UP
novel-m0040-3p	1.448933074	0.035836655	UP
novel-m0041-3p	1.448933074	0.035802396	UP
novel-m0042-3p	1.448933074	0.035755084	UP
novel-m0078-5p	1.407254102	0.046943548	UP

**Table 5:** DEmRNAs.

**Id**	**Log2 (FC)**	***P*-value**	**Symbol**	**Regulation**
ENSG00000283149	−9.373590215	0.048177772	–	DOWN
ENSG00000285238	−9.120669887	0.047017499	–	DOWN
ENSG00000196826	−6.594946589	0.005950249	–	DOWN
ENSG00000256407	6.196397213	0.020316197	–	UP
ENSG00000120903	5.984893108	0.015972752	CHRNA2	UP
ENSG00000269693	5.824428435	0.044393428	–	UP
ENSG00000243708	4.14974712	0.006466558	PLA2G4B	UP
ENSG00000268400	3.857980995	0.000344882	–	UP
ENSG00000180574	−3.64385619	0.044530877	EIF2S3B	DOWN
ENSG00000203618	−3.087462841	0.04459388	GP1BB	DOWN
ENSG00000258644	2.669278787	0.011001883	SYNJ2BP-COX16	UP
ENSG00000286237	2.662965013	0.037227607	ARMCX5-GPRASP2	UP
ENSG00000285130	−2.169925001	0.007819973	–	DOWN
ENSG00000285932	−2.073248982	0.00788196	–	DOWN
ENSG00000081853	1.874469118	0.023777064	PCDHGA2	UP
ENSG00000288710	1.811238357	0.028399634	–	UP
ENSG00000205572	−1.739685096	0.031078684	SERF1B	DOWN
ENSG00000224420	1.490050854	0.034327488	ADM5	UP
ENSG00000285953	−1.301790449	0.017034279	–	DOWN
ENSG00000180071	1.206450877	0.038552507	ANKRD18A	UP
ENSG00000270106	−1.108059746	0.014049763	TSNAX-DISC1	DOWN
ENSG00000128917	1.069041644	0.036951898	DLL4	UP
ENSG00000099864	0.951905712	0.036496947	PALM	UP
ENSG00000105655	0.932095935	0.046649885	ISYNA1	UP
ENSG00000254206	0.923131237	0.00536801	NPIPB11	UP
ENSG00000286106	0.918699898	0.033431328	Notch2NLR	UP
ENSG00000285253	0.861616516	0.036903938	–	UP
ENSG00000268350	0.773882089	0.018657013	FAM156A	UP
ENSG00000265118	−0.683517083	0.045119276	–	DOWN
ENSG00000178163	−0.597673552	0.045106597	ZNF518B	DOWN

### GO and KEGG pathway enrichment analysis of DEmRNAs and DEcircRNAs

GO, an internationally recognized system for classifying gene functions, comprises three ontologies elucidating molecular function, cellular component, and biological processes, which provide a comprehensive understanding of gene attributes and their roles in living organisms. In living systems, genes collaborate to perform various biological functions. To uncover the potential functions of DEmRNAs and DEcircRNAs, we conducted GO annotation and KEGG pathway enrichment analyses on the parent genes of 371 DEcircRNAs (204 upregulated and 167 downregulated) and 30 DEmRNAs (18 upregulated and 12 downregulated), allowing us to gain deeper insights into their roles in biological processes and molecular pathways.

**Figure 3:**
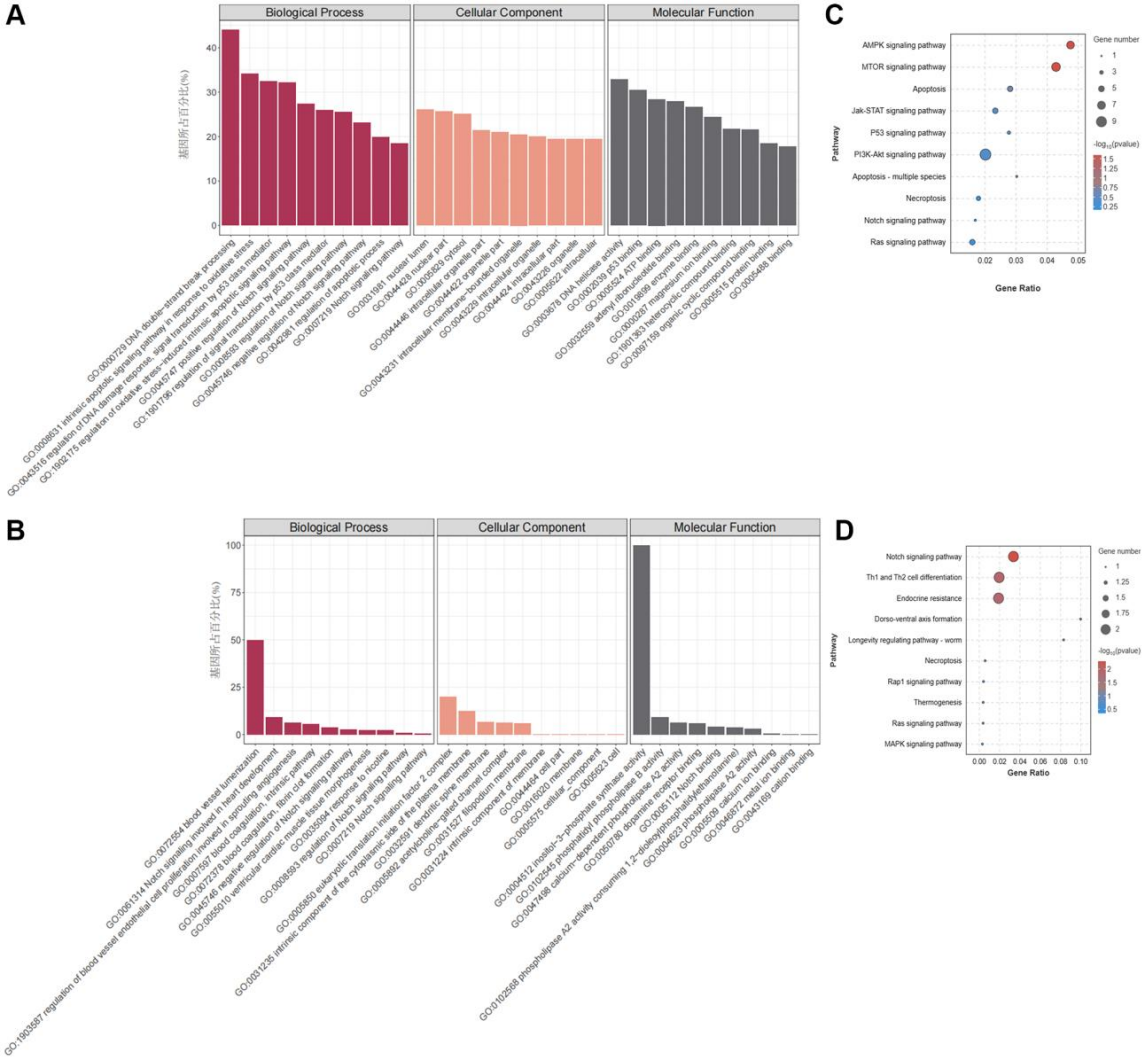
**GO and KEGG pathway enrichment analysis of DEmRNAs and DEcircRNAs.** (**A**) GO enrichment analysis of DEcircRNA parental genes. (**B**) GO enrichment analysis of DEmRNAs. (**C**) KEGG enrichment analysis of DEcircRNA parent genes. (**D**) KEGG enrichment analysis of DEmRNAs.

Figure 3A, 3C present the enrichment analysis results for the GO and KEGG pathways associated with the parent genes of the DEcircRNAs. Annotation analysis revealed that the parent genes of DEcircRNAs are predominantly located in the cytoplasm and are involved in significantly enriched biological processes related to apoptosis, such as DNA double-strand break processing and the regulation of oxidative stress. KEGG pathway enrichment analysis revealed that the parent genes of DEcircRNAs are primarily enriched in various apoptosis-related signalling pathways, including the Notch signalling pathway, the AMPK signalling pathway, and the mTOR signalling pathway.

According to the GO annotations of the DEmRNAs (Figure 3B), there was a notable abundance of biological processes related to CVD, including blood vessel recruitment, the involvement of the Notch signalling pathway in heart development, the regulation of blood vessel endothelial cell promotion involved in angiogenesis, and other processes. KEGG pathway enrichment analysis revealed that DEmRNAs were primarily involved in regulating cell death patterns, cell differentiation signalling pathways, and apoptosis signalling pathways, such as the Notch signalling pathway (Figure 3D). The above GO and KEGG enrichment analysis results suggest that nicotine-induced macrophage apoptosis may be related to the activation of the Notch signalling pathway.

### Construction of the circRNA-miRNA–mRNA regulatory network

**Figure 4:**
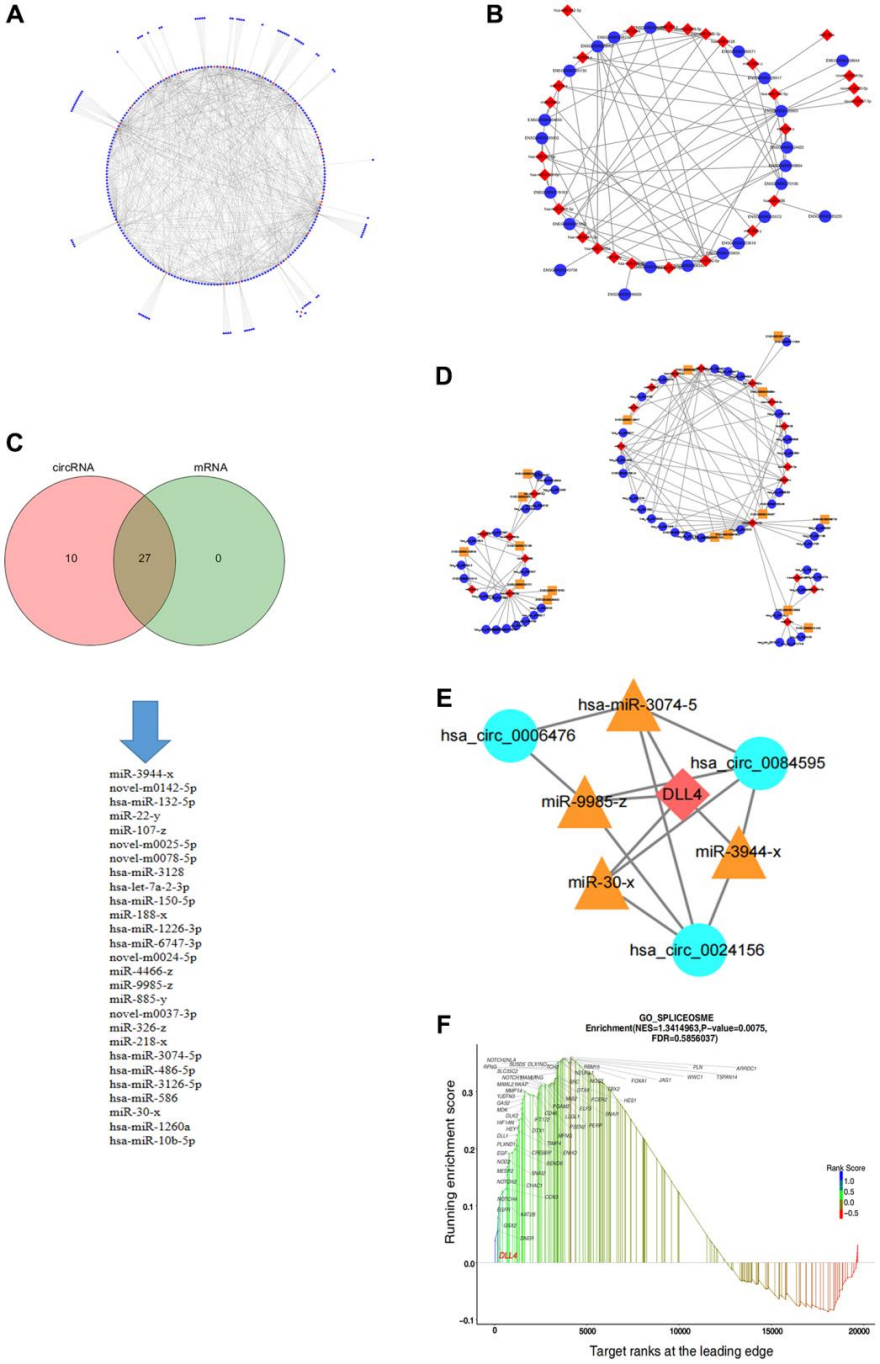
**Construction of the circRNA-miRNA–mRNA regulatory network in apoptotic cells.** (**A**) circRNA-miRNA regulatory network. (**B**) miRNA–mRNA regulatory network. (**C**) Intersection of miRNA targets of circRNAs and mRNAs. (**D**) ceRNA regulatory network. (**E**) ceRNA regulatory network targeting *DLL4*. (**F**) GSEA of genes related to the notch signalling pathway.

The ceRNA theory has emerged as a novel mechanism for RNA interactions. In recent years, this topic has garnered substantial attention across various research domains, including CVD investigations, pathological studies, and developmental research. To predict potential miRNA targets among all DEcircRNAs and DEmRNAs, we used the SCC to assess miRNA targeting for the 371 DEcircRNAs and 30 DEmRNAs, respectively, which led to the construction of interaction networks for DEcircRNA-DEmiRNA and DEmRNA-DEmiRNA pairs (Figure 4A, 4B). Furthermore, we established a comprehensive circRNA-miRNA–mRNA network integrating circRNA-miRNA and miRNA–mRNA pairs based on shared miRNA binding sites (Figure 4C), resulting in the development of a precise regulatory network involving 30 circRNAs, 19 miRNAs, and 14 mRNAs, which was visualized via Cytoscape (Figure 4D). Within the ceRNA network comprising 14 mRNAs, we focused on *DLL4*, a key component in apoptosis progression, following which we constructed a ceRNA network targeting *DLL4*, encompassing three circRNAs and four miRNAs (Figure 4E). GSEA can explore gene signalling pathways that affect related diseases at the RNA level, providing direct evidence for specific basic experiments. To elucidate the functional significance of the gene sets between the two experimental groups, we employed GSEA, which identified gene sets that exhibited coordinated differences in the expression matrix of all genes. We considered genes with subtle variations. Notably, GSEA showed that compared with those in the control group, nicotine-treated macrophages exhibited significant upregulation of the Notch signalling pathway, which is closely related to cell apoptosis. In addition, the expression of the Notch ligand *DLL4* significantly increased in this pathway (Figure 4F).

### Nicotine-induced apoptosis via DLL4 in THP-1 macrophages

**Figure 5:**
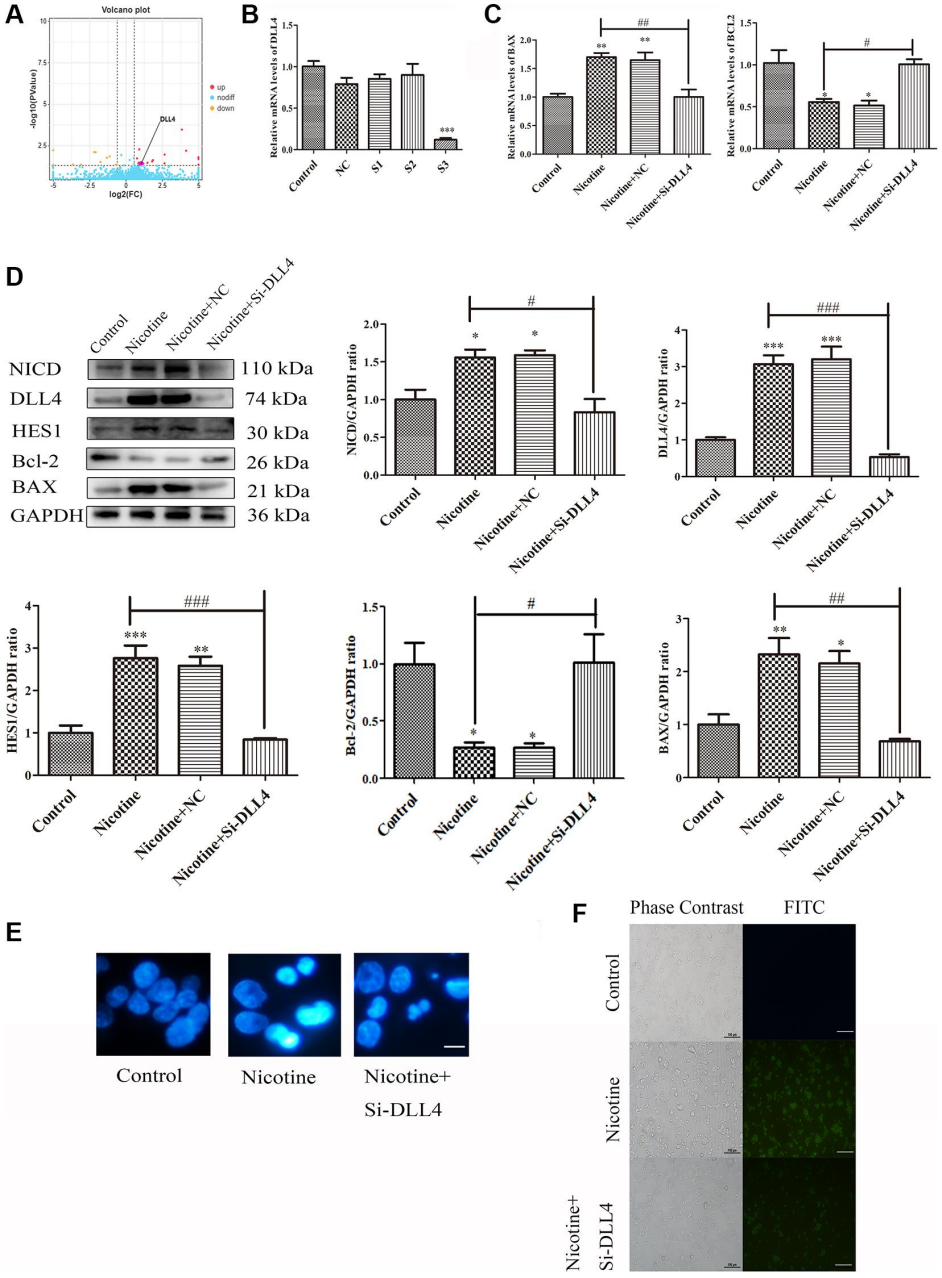
**Nicotine activates apoptosis through the DLL4/Notch signalling pathway.** (**A**) Volcano map depicting *DLL4* expression. (**B**) Representative qPCR results illustrating the downregulation of *DLL4* upon siRNA-mediated *DLL4* knockdown. (**C**) qPCR analysis of the impact of *DLL4* knockdown on apoptosis-related genes. (**D**) WB analysis showing the effects of *DLL4* knockdown on apoptosis and Notch signalling pathway-related proteins. (**E**) Hoechst staining of THP-1 macrophages (bright blue represents the apoptotic cell nucleus) (scale bar: 50 μm). (**F**) TUNEL assay to assess apoptosis-positive THP-1 macrophages (green fluorescence indicates apoptotic cells) (scale bar: 50 μm). (*n* = 3. The data are presented as the mean ± SD. ^*^P < 0.05, ^**^P < 0.01 and ^***^P < 0.001 vs. the control group; ^#^*P* < 0.05, ^##^*P* < 0.01 and ^###^*P* < 0.01 vs. the nicotine group).

The DLL4/Notch signalling pathway is a crucial signal transduction system that regulates cellular processes such as proliferation, differentiation, apoptosis, invasion and metastasis. Abnormal regulation of this pathway contributes significantly to the development of various CVDs [20]. Volcano plots showed elevated *DLL4* expression in THP-1 macrophages treated with nicotine (Figure 5A). To explore the relationship between apoptosis and the DLL4/Notch signalling pathway, we knocked down *DLL4* using siRNA and selected the S3 interference fragment (*P* < 0.001) (Figure 5B). Knocking down *DLL4* reversed nicotine-induced macrophage apoptosis while reducing the expression of the proapoptotic gene BAX (*P* < 0.01) and increasing the expression of the antiapoptotic gene *BCL2* (*P* < 0.05) (Figure 5C). The ligand DLL4 of the Notch signalling pathway is critical for regulating the germination and branching morphology of blood vessels. WB analysis was used to determine the expression levels of critical proteins in the Notch signalling pathway, including DLL4, NICD, and HES1, which were significantly inhibited after DLL4 interference (*P* < 0.05). This indicates that interference with DLL4 inhibits nicotine-induced Notch signalling pathway activation (Figure 5D). The Hoechst staining results showed that nicotine-treated THP-1 macrophages exhibited characteristic concentrated bright nuclei of apoptotic cells, which significantly disappeared after *DLL4* interference (Figure 5E). The Hoechst staining results again emphasized the inhibitory effect of *DLL4* interference on nicotine-induced apoptosis. TUNEL staining further confirmed these findings, showing that nicotine treatment increased the number of apoptotic macrophages. At the same time, *DLL4* interference significantly reduced the number of apoptotic cells among macrophages treated with nicotine (Figure 5F). This indicates that *DLL4* is a crucial gene in nicotine-induced macrophage apoptosis. These results prove that nicotine activates macrophage apoptosis through the DLL4/Notch signalling pathway.

### *Circ_0006476* regulates THP-1 macrophage apoptosis

**Figure 6:**
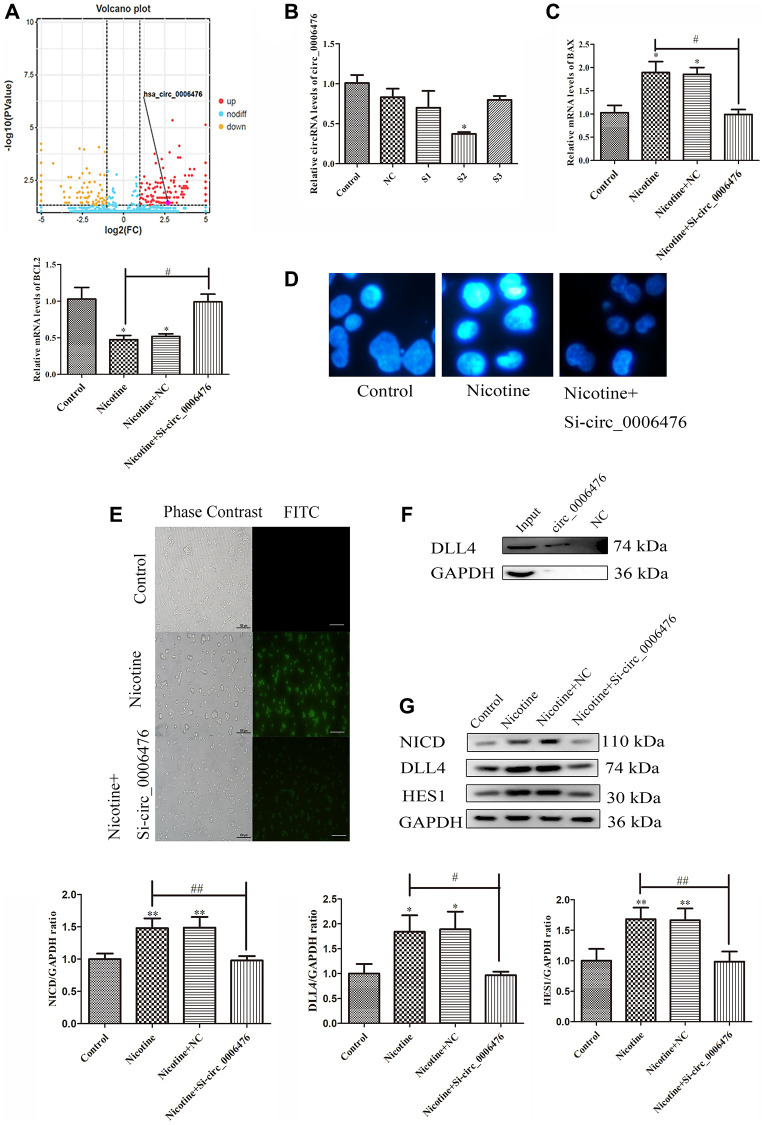
***Circ_0006476* participates in nicotine-induced apoptosis.** (**A**) Volcano plot displaying *circ_0006476* expression. (**B**) Representative qPCR results indicating the reduced expression of *circ_0006476* following siRNA-mediated *circ_0006476* knockdown. (**C**) qPCR analysis of the impact of *circ_0006476* knockdown on the expression of apoptosis-related genes. (**D**) Hoechst staining of THP-1 macrophages (bright blue represents the apoptotic cell nucleus) (scale bar: 50 μm). (**E**) TUNEL assay to assess apoptosis-positive THP-1 macrophages (green fluorescence indicates apoptotic cells) (scale bar: 50 μm). (**F**) The interaction between *circ_0006476* and DLL4 was detected by WB combined with RNA pulldown. (**G**) WB analysis showing the effects of *circ_0006476* knockdown on apoptosis and Notch signalling pathway-related proteins. (*n* = 3. The data are presented as the mean ± SD. ^*^P < 0.05 and ^**^P < 0.01 vs. the control group; ^#^*P* < 0.05 and ^##^*P* < 0.01 vs. the nicotine group).

Previous studies have confirmed the significant regulatory role of circRNAs in apoptosis. Within the ceRNA network targeting *DLL4*, which includes three circRNAs, we focused on *hsa_circ_0006476*. Volcano plots revealed elevated expression of *circ_0006476* in nicotine-treated THP-1 macrophages (Figure 6A). Subsequently, we used qPCR to screen for the optimal interfering fragment of *circ_0006476*, selected the S2 fragment with the best interfering effect (*P* < 0.05) (Figure 6B), and evaluated the expression of apoptosis-related genes after *circ_0006476* interference. Compared with that in nicotine-treated cells, the expression of the proapoptotic gene BAX in nicotine-treated macrophages was significantly inhibited (*P* < 0.05) after *circ_0006476* interference; however, the antiapoptotic gene *BCL2* was significantly activated (*P* < 0.05) in nicotine-treated macrophages after *circ_0006476* interference (Figure 6C). Similarly, the results of Hoechst (bright blue represents the apoptotic cell nucleus) and TUNEL staining (green fluorescence represents apoptotic cells) further demonstrated that inhibiting *circ_0006476* expression effectively reversed nicotine-induced apoptosis (Figure 6D–6E). Then, we performed RNA pull-down analysis using the *circ_0006476* probe and found that compared with the negative control, DLL4 strongly bound to *circ_0006476*. This indicates that *circ_0006476* is involved in the regulation of DLL4 expression (Figure 6F). Finally, we confirmed whether *circ_0006476* is involved in the Notch signalling pathway in cell apoptosis. Therefore, we detected the expression levels of Notch signalling pathway-related proteins after *circ_0006476 interference*. The WB results showed that after interfering with *circ_0006476*, the expression of the Notch signalling pathway proteins NICD, DLL4, and HES1 was significantly reduced (Figure 6G). These findings conclusively establish the involvement of *circ_0006476* in nicotine-induced THP-1 macrophage apoptosis.

### *Circ_0006476* acts as a miR-3074-5p sponge to regulate DLL4 expression in THP-1 macrophages

**Figure 7:**
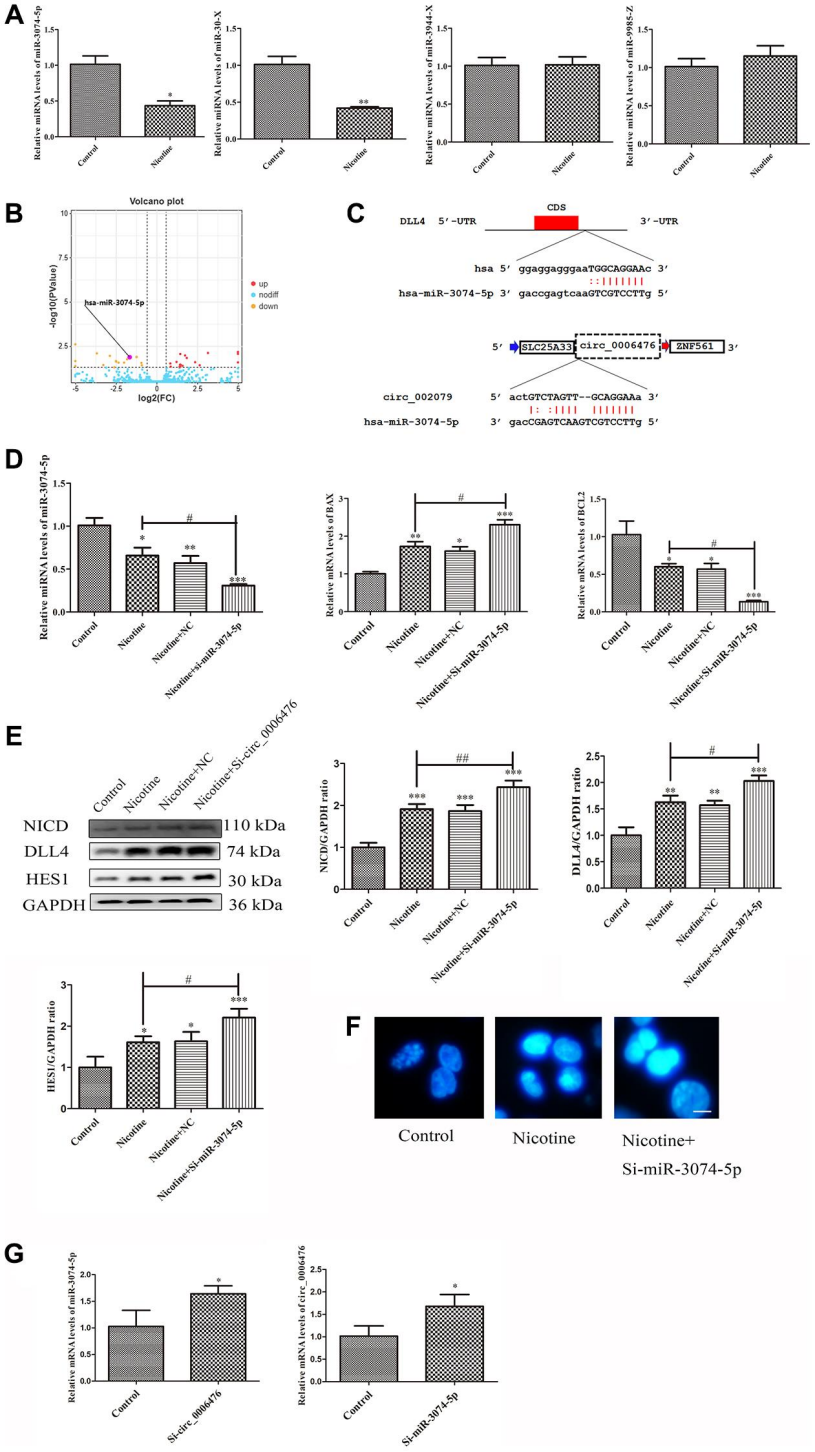
***Circ_0006476*/*miR-3074-5p* sponge adsorption function.** (**A**) qPCR analysis of DEmiRNA expression levels. (**B**) Volcano plot displaying *miR-3074-5p* expression. (**C**) The predicted binding sites of *DLL4* and *circ_0006476* on *miR-3074-5p*. (**D**) qPCR assessment of the impact of *miR-3074-5p* knockdown on *miR-3074-5p* and apoptosis-related genes. (**E**) WB analysis showing the effects of *miR-3074-5p* knockdown on apoptosis and Notch signalling pathway-related proteins. (**F**) Hoechst staining of THP-1 macrophages (bright blue represents the apoptotic cell nucleus) (scale bar: 50 μm). (**G**) Verification of the sponge adsorption relationship between *circ_0006476* and *miR-3074-5p.* (*n* = 3. The data are presented as the mean ± SD. ^*^P < 0.05, ^**^P < 0.01 and ^***^P < 0.001 vs. the control group; ^#^*P* < 0.05 and ^##^*P* < 0.01 vs. the nicotine group).

As circRNAs are considered to function primarily competitively by sequestering functional miRNAs and influencing gene expression, we investigated the potential regulatory effect of miRNAs associated with *circ_0006476* on DLL4 expression. Our ceRNA network analysis revealed that *circ_0006476* harbors binding sites for *miR-3074-5p*, *miR-30x*, *miR-3944-x* and *miR-9985-z*, among which *miR-3074-5p* is a known miRNA. To explore the role of these miRNAs in nicotine-induced apoptosis, we conducted qPCR to assess their expression after nicotine treatment, which revealed a significant upregulation of *miR-3074-5p* (Figure 7A). Volcano plots further confirmed the elevated expression of *miR-3074-5p* in THP-1 macrophages exposed to nicotine (Figure 7B). To further confirm whether *miR-3074-5p* is involved in the regulation of DLL4 by *circ_0006476*, we used miRanda and TargetScan for target gene prediction. The miRNA target gene prediction results were the intersection of the target gene prediction results obtained by the two methods (Figure 7C). This indicates that *miR-3074-5p* is likely involved in the regulation of DLL4 by *circ_0006476* in nicotine-induced macrophages. Further validation is needed to determine whether *miR-3074-5p* is involved in the regulation of *DLL4* by *circ_0006476* in nicotine-induced macrophages. Subsequently, after interfering with *miR-3074-5p expression*, the expression of apoptotic genes was detected using qPCR. Compared with nicotine treatment, *miR-3074-5p* interference significantly increased the expression of the proapoptotic gene *BAX* in nicotine-treated macrophages (*P* < 0.05). After *miR-3074-5p* interference, the expression of the antiapoptotic gene *BCL2* in nicotine-treated macrophages was significantly reduced (*P* < 0.05) (Figure 7D). We detected apoptosis and the expression of Notch signalling pathway-related proteins after interfering with *miR-3074-5p* and determined the impact of *miR-3074-5p* on the Notch signalling pathway. The WB results revealed that inhibition of *miR-3074-5p* expression activated the Notch signalling pathway and upregulated apoptosis (Figure 7E). These findings are consistent with the results from Hoechst (bright blue represents the apoptotic cell nucleus) (Figure 7F), collectively supporting the role of *circ_0006476* as a *miR-3074-5p* sponge, thereby regulating DLL4 expression and apoptosis in THP-1 macrophages. The above results indicate that *miR-3074-5p* may be involved in the regulation of DLL4 by *circ_0006476* in nicotine-induced macrophages, but the manner in which it participates is not yet clear. Then, we detected changes in the gene expression of *miR-3074-5p* in nicotine-treated macrophages after interference with *circ_0006476*. After interference with *miR-3074-5p*, changes in the gene expression of *circ_0006476* were detected. The qPCR results revealed a sponge effect between the two, manifested as a significant increase in the gene expression of *miR-3074-5p* after interference with *circ_0006476* (*P* < 0.05) and a significant increase in the gene expression of *circ_0006476* after interference with *miR-3074-5p* (*P* < 0.05) (Figure 7G). All the above results collectively confirmed that *circ_0006476*-*miR-3074-5p* is involved in the activation of DLL4 and apoptosis in nicotine-induced macrophages through sponge adsorption.

## DISCUSSION

THP-1 (human monocytic leukemia) cells are derived from the blood of patients with acute monocytic leukemia. In experimental studies, it is often induced to differentiate into macrophages and foam cells and is usually applied in *in vitro* mechanistic research on AS. There are three main reasons for this: first, macrophages are crucial immune effector cells that play essential roles in innate and adaptive immune responses. Macrophages are among the most active immune cells in atherosclerosis. They can induce inflammation by engulfing and decomposing low-density lipoprotein on the surface of vascular endothelial cells [21]. Moreover, it is also a highly heterogeneous cell type that can be activated into different types and exhibit different phenotypes and functions under the influence of the local microenvironment. Due to the heterogeneity of macrophages in different tissue environments and physiological and pathological conditions, macrophages are divided into two main types: M1 macrophages and M2 macrophages [22]. In addition, after THP-1 cells are induced to differentiate into macrophages, they can transform into foam cells by taking up a large amount of lipids, which is also the cause of AS [23].

Although significant progress has been made in the treatment of AS, the precise mechanisms underlying its onset and progression remain elusive, particularly in the context of circular RNA (circRNA). Therefore, there is a pressing need to elucidate the molecular mechanisms driving the development of AS and identify effective biomarkers that could improve the treatment and prognosis of AS patients. In recent years, the competitive endogenous RNA (ceRNA) network hypothesis has attracted the attention of researchers, who have established a link between protein-coding mRNAs and noncoding RNAs. According to the ceRNA hypothesis, circRNAs can function as miRNA sponges to influence downstream mRNA expression [24–25]. Numerous studies have shown the pivotal role of circRNAs and their mediated ceRNA regulatory networks in the pathogenesis and progression of various CVDs [16]. Hence, this study aimed to elucidate the potential mechanisms involving circRNA-related ceRNA networks in the emergence and progression of AS. Our findings revealed that these ceRNA regulatory networks contribute to maintaining the homeostasis of both the cardiac and vascular systems, as well as the pathophysiology of CVDs, making them promising tools for the diagnosis, prognosis and treatment of diverse CVDs.

To date, there has been a lack of comprehensive investigations into the expression patterns of circRNAs that are differentially expressed between nicotine-induced apoptotic cells and their nonapoptotic counterparts. Thus, the present study aimed to fill this knowledge gap by systematically analysing circRNA expression in cells undergoing nicotine-induced apoptosis using advanced sequencing technology. When compared with the circBase database, most of the identified circRNAs were found to be previously unreported. According to our predetermined selection criteria (|FC|>2, *P* < 0.05), we identified a total of 371 DEcircRNAs, 204 of which exhibited increased expression and 167 of which exhibited decreased expression. Additionally, we assessed differential mRNA expression in nicotine-treated cells using paired comparisons between control and nicotine-treated cells and identified 30 DEmRNAs, 18 of which were upregulated and 12 of which were downregulated. Interestingly, a significant proportion of DEcircRNAs (55%) and DEmRNAs (60%) exhibited an upregulated pattern, indicating a consistent expression trend between circRNAs and mRNAs.

GO enrichment and KEGG pathway analyses revealed the functional relevance of parental genes associated with DEcircRNAs. These genes are significantly enriched in apoptosis-related pathways, such as the Notch, AMPK and mTOR signalling pathways. Subcellular localization analysis (GO-CC) revealed the predominant cytoplasmic localization of the parental genes of the DEcircRNAs, which is in line with the known cytoplasmic role of exonic circRNAs in posttranscriptional regulation. Similarly, DEmRNAs in apoptotic cells, as revealed by GO and KEGG analyses, participate in cell death patterns, cell differentiation signalling and apoptosis pathways, including the Notch pathway. Collectively, these findings underscore the crucial roles of DEcircRNAs and DEmRNAs in apoptosis-related pathways. Furthermore, they suggested that the circRNA-based ceRNA network may contribute significantly to macrophage apoptosis initiation and progression.

**Figure 8:**
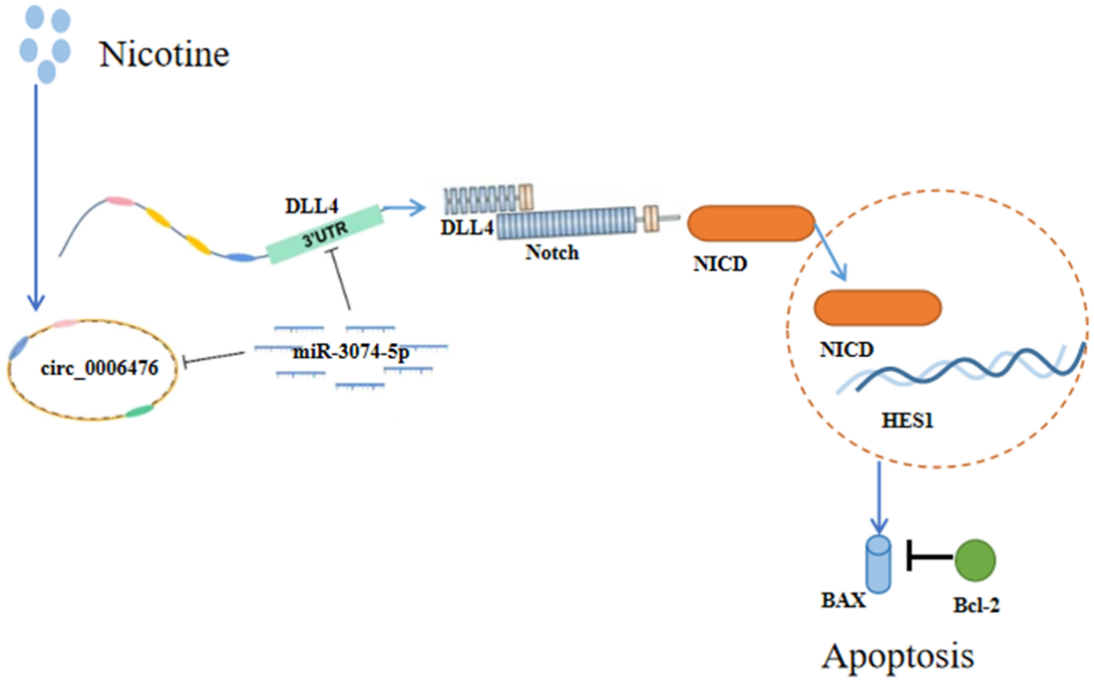
**Schematic representation of the effects of nicotine on THP-1 macrophages.** Nicotine exposure upregulates *circ_0006476* expression, leading to competitive binding with *miR-3074-5p* and subsequent activation of *DLL4*. Activated DLL4 triggers apoptosis through the Notch signalling pathway.

An increasing number of studies have highlighted the role of circRNAs as miRNA sponges that indirectly regulate gene expression. To elucidate the precise functions and mechanisms of DEcircRNAs in apoptosis, we constructed a circRNA-miRNA–mRNA regulatory network comprising 31 circRNAs, 19 miRNAs, and 14 mRNAs. To gain insights into the potential pathways and biological functions of mRNA targets within ceRNA networks underlying apoptosis, we performed GSEA, focusing on gene sets instead of individual genes. The GSEA results revealed significant activation of the Notch signalling pathway in nicotine-treated macrophages, with significant upregulation of DLL4, a key player in this pathway. Next, we constructed a ceRNA regulatory network focusing on DLL4 involving three circRNAs and four miRNAs. For molecular validation, we selected one of these ceRNA networks, namely, *circ_0006476*-*miR-3074-5p*-*DLL4*. Our experimental findings collectively support the notion that 1 μM nicotine orchestrates *circ_0006476*-miR-3074-5p-DLL4 interactions to activate the Notch signalling pathway, triggering apoptosis in THP-1 macrophages and thereby accelerating the onset and progression of CVD (Figure 8). Overall, this study offers novel insights into AS pathogenesis and proposes potential regulatory mechanisms warranting further investigation.

This study has several limitations that need to be addressed. Although we established the first circRNA expression profile in nicotine-induced macrophages, it is important to note that we did not conduct *in vivo* experimental validation, which should be performed on a larger scale in the future. Additionally, it is worth considering that genetic factors influence CVD, and nicotine is not the only harmful component present in smoking. Consequently, variations in the ceRNA network may be observed in both *in vivo* and *in vitro* experiments. At present, research related to circRNAs is still in its early stages. In the future, it remains to be further explored whether circRNAs bind to proteins, serve as translation templates, and regulate gene transcription in the occurrence and development of CVD. In addition, more basic research and clinical trials are needed to explore the relevant mechanisms of circRNA regulation of CVD. With further research, the early prevention, diagnosis, and treatment of CVD are expected to improve to the molecular level, or abnormal expression of circRNAs can be quickly screened through gene chips, improving the early detection rate of CVD and providing a new perspective for the early diagnosis and treatment of CVD by targeting circRNAs.

## CONCLUSION

Our experimental findings collectively support the notion that 1 μM nicotine orchestrates *circ_0006476*-*miR-3074-5p*-*DLL4* interactions to activate the Notch signalling pathway, triggering apoptosis in THP-1 macrophages and thereby accelerating the onset and progression of CVD (Figure 8).
